# Elimination of Ox-LDL through the liver inhibits advanced atherosclerotic plaque progression

**DOI:** 10.7150/ijms.63065

**Published:** 2021-09-09

**Authors:** Zhiwen Wang, Xiaopeng Guo, Qing Zhang, Gaohui Du, Zhuanglin Zeng, Chuansheng Zheng, Yumiao Wei

**Affiliations:** 1 Department of Cardiology, Union Hospital, Tongji Medical College, Huazhong University of Science and Technology, Wuhan, China.; 2 Department of Radiology, Union Hospital, Tongji Medical College, Huazhong University of Science and Technology, Wuhan, China.; 3 Department of Emergency Medicine, Union Hospital, Tongji Medical College, Huazhong University of Science and Technology, Wuhan, China.

**Keywords:** Ox-LDL, LOX-1, Atherosclerosis, Foam Cell, Necrotic Core

## Abstract

**Aim:** In the late stage of atherosclerosis, the endothelial barrier of plaque is destroyed. The rapid deposition of oxidized lipids in the circulation leads to migration of numerous smooth muscle cells and macrophages, as well as foaming necrosis. The plaque progresses rapidly, and vulnerable plaques can easily induce adverse cardiovascular events. Here, we take the principle of gene editing to transfer the liver to express the LOX-1 receptor which is more sensitive to Ox-LDL by using AAV8 containing a liver-specific promoter. In this way, we want to explore whether the progress of advanced atherosclerosis and the stability of advanced plaque can be improved when the liver continues to clear Ox-LDL from the circulation.

**Methods and Results:** In order to explore the effect of the physiological and continuous elimination of Ox-LDL through the liver on advanced atherosclerosis, we chose ApoE^-/-^ mice in high-fat diet for 20 weeks. After 16 weeks of high-fat diet, the baseline group was sacrificed and the specimens were collected. The virus group and the control group were injected with the same amount of virus dilution and normal saline through the tail vein, and continued to feed until 20 weeks of high-fat diet, and then sacrificed to collect specimens. The results showed that LOX-1 was ectopically and functionally expressed in the liver as an Ox-LDL receptor, reducing the content of it in circulation. Compared with the control group, the degree of plaque progression in the virus group was significantly reduced, similar to the baseline group, the plaque necrosis core decreased, and the collagen fiber content increased. In addition, there are more contractile smooth muscle cells in the plaques of the virus group instead of synthetic ones, and the content of macrophages was also reduced. These data suggested that the virus group mice have greatly increased advanced plaque stability compared with the control group mice.

**Conclusions:** Due to the destruction of endothelial barrier in advanced plaques, rapid deposition of Ox-LDL can result in fast plaque progression, increased necrotic cores, and decreased stability. Our research shows that the use of AAV8 through gene editing allows the liver to express LOX-1 receptors that are more sensitive to Ox-LDL, so that it can continue to bind Ox-LDL in the circulation and exploit the liver's strong lipid metabolism ability to physiologically clear Ox-LDL, which can inhibit the rapid progress of advanced plaque and increase the stability of plaque.

## Introduction

Cardiovascular events caused by atherosclerosis are currently the leading cause of death worldwide. Over the last century, atherosclerotic research has provided us with a basic direction and strategy for treating the disease. However, no effective interventions for advanced atherosclerotic lesions exists 1. In clinical treatment, most patients with acute coronary syndromes are only discovered after the onset of severe symptoms. At this time, atherosclerosis has progressed to advanced or even terminal stages 2, making highly targeted treatment measures challenging to implement, resulting in cardiovascular death. A clinical study demonstrated that lipid-lowering and antithrombotic therapy in the early stage of atherosclerosis could maintain the patient's stability for about three years, but for patients with advanced atherosclerosis, current treatment measures cannot prevent cardiovascular death 3. Therefore, the study of appropriate treatment strategies for advanced atherosclerosis has great clinical significance.

In the late stage of atherosclerotic lesions, the endothelial barrier is almost completely destroyed, and a large amount of oxidized modified LDL is deposited under the vascular intima. These deposited Ox-LDL attract macrophages to continue phagocytosis and secrete proteolytic enzymes until apoptosis and necrosis, forming a soft and unstable lipid-rich cavity containing cholesterol necrotic crystals and devoid of supporting collagen and cells, referred to as the "necrotic core" 4, 5. In addition, fibrocytes form a fibrous cap between the necrotic core and the lumen. In the late stage, pro-inflammatory macrophages secrete matrix metalloproteinases and other related degradation molecules, as well as the foam and apoptosis of smooth muscle cells that secrete the extracellular matrix of the core component of the fibrous cap cause the fiber cap to become extremely thin 6. Therefore, huge lipid necrotic core and extremely thin fibrous cap eventually lead to plaque rupture and thrombus formation, resulting in acute coronary syndromes such as acute myocardial infarction, sudden coronary heart disease death, and unstable angina pectoris 7. According to studies, thrombus accounts for 60% of sudden deaths caused by atherosclerosis, and 55%-60% of thrombus is caused by late-stage vulnerable plaque rupture 8. Therefore, in the late stage of atherosclerosis, in addition to lowering lipids, the primary goal should be to treat stable plaque.

Studies have shown that the occurrence and development of atherosclerosis are closely related to lipid metabolism disorders 9. It is prominent that low-density lipoprotein (LDL) particles, which are mainly loaded with cholesterol, cannot be fully absorbed and metabolized by the liver and deposited on the endothelial cells of the arterial vascular wall. However, the vascular endothelial cells do not have the ability to physiologically metabolize LDL, so the deposited LDL is further oxidized and modified as Ox-LDL. The emergence of the latter activates the innate immune system represented by macrophages, triggering a chronic inflammatory response in the vascular wall. But the phagocytosis of Ox-LDL by macrophages cannot completely degrade it. With the accumulation of lesions, a large number of macrophages foamed in the late stage of atherosclerosis 10. At the same time, the excessive accumulation of Ox-LDL further induced smooth muscle cells to transform into macrophages and then foamed 11. As a result, we can demonstrate that Ox-LDL has a detrimental effect on the entire process of atherosclerosis formation. There have been clinical studies on the antioxidant treatment of Ox-LDL in the early stage of the disease, such as Vitamin C, etc. but the effect is non-significant 12. In addition, there are few non-antioxidant therapies for Ox-LDL in the late stages of the disease. Accordingly, we propose whether clearing the circulating Ox-LDL in the advanced atherosclerosis can reduce necrotic core, stabilize plaque, and inhibit the rapid progress of advanced atherosclerosis.

Lectin-like oxidized low-density lipoprotein receptor-1 (LOX-1) is a major receptor that recognizes and internalizes Ox-LDL in the circulation. It belongs to the scavenger receptor family and mainly exists in endothelial cells, macrophages, smooth muscle cells, etc. 13, 14, which can sensitively identify and combine with LDL that is only slightly oxidized 15. Under physiological conditions, the LOX-1 expression is very low. However, when oxidative stress or inflammation occurs in the body, its expression will increase rapidly. Ox-LDL is the most effective activator of LOX-1, or Ox-LDL is the main specific ligand of LOX-1 receptor. Ox-LDL is recognized and swallowed by vascular endothelial cells via the LOX-1 receptor, causing endothelial cell dysfunction and related damage. What's more, the LOX-1 receptor is more specific to Ox-LDL, and can play a role when LDL particles are slightly oxidized. Therefore, for eliminating Ox-LDL, LOX-1 may be a better choice 16.

As we all know, the liver has a highly complete metabolic system and is also the center for lipid metabolism and transformation. However, the huge number of lipid metabolism factory hepatocytes cannot play its important advantage in the clearance of Ox-LDL only due to the lack of corresponding receptors. Therefore, we envisioned that, in view of the current mature gene editing technology, using AAV8 containing a liver-specific promoter to transfect the liver to make the hepatocytes express LOX-1. With its powerful lipid metabolism capacity, the liver continuously removes the Ox-LDL in the circulation of advanced atherosclerosis to inhibit the progression of advanced atherosclerosis and stabilize the advanced plaque.

Here, we found that the unstable plaque formed rapidly in the advanced atherosclerosis. Through the use of gene editing technology to make hepatocytes express LOX-1 ectopically, the liver can continuously clear the Ox-LDL accumulated in the circulation of advanced atherosclerosis, and finally inhibit the fast development of advanced atherosclerosis and occurrence of plaque rupture. Hence, we believe that our study provides a novel treatment method for the treatment of advanced atherosclerosis in addition to lowering lipids, which has great clinical significance.

## Materials and Methods

The design, packaging, and amplification of AAV8-LOX-1 adeno-associated virus vector was assisted by GeneChem Co., Ltd (Shanghai, China).

### Animals

All the procedures involving animal experiments were approved by the Ethics Committee of Union Hospital, Huazhong University of Science and Technology, China, and were conducted in accordance with the National Institutes of Health (NIH) Guide for the Care and Use of Laboratory Animals.

Seven-week-old male apolipoprotein E (ApoE) knockout mice were purchased from Vital River Co., Ltd (Beijing, China) and housed in a single-cage single-chamber system at the specific-pathogen-free (SPF) grade Animal Experimental Center, Tongji Medical College, Huazhong University of Science and Technology. After a week of normal diet feeding, the mice have adapted to the environment, they were randomly divided into three groups: baseline group (n=10), virus group (n=10), and control group (n=10). After 16 weeks of high-fat feeding (high-fat feeding started at eight weeks of age), mice in the baseline group were sacrificed, and corresponding specimens were collected to test various indicators. Simultaneously, the virus group was injected with virus dilution, and the control group was injected with normal saline. High-fat diet feeding for another 4 weeks later, the mice of the two groups were sacrificed and corresponding specimens were collected.

Intervention protocol: The mice of three groups were fed a high-fat diet for 16 weeks without any treatment. After that, the AAV8-LOX-1 adeno-associated virus vector with a virus titer of 1.16×10^13^ vg (virus genomes)/mL was diluted with sterile PBS buffer to 1.16×10^11^ vg/piece/100 μL, prepared for immediate use and stored in an ice box to avoid repeated freezing and thawing affecting the virus titer. The virus group was injected with 1.16×10^11^ vg/mouse/100 μL of AAV8-LOX-1 through the tail vein of the mice, and the control group was injected with 100 μL of sterile normal saline. The baseline group mice were sacrificed to obtain materials.

### Analysis of Dil-labelled Ox-LDL (Dil-Ox-LDL) uptake

After injecting Dil-Ox-LDL via the tail vein, cryosectioning of liver tissue was performed. Laser scanning confocal microscopy was used to detect the red-fluorescence (Dil-Ox-LDL) in liver sections. The specific steps were as follows: The mice from viral and control groups were injected 30 μg/100 μL Dil-Ox-LDL (freshly prepared and protected from light during injection) into the tail vein. Thirty minutes after the Dil-Ox-LDL injection, the mice were sacrificed and perfused with pre-cooled saline from heart, and then the liver was collected. As for the cryosections, we chose 10μm thickness of each liver section and 8 specimens were obtained from each liver sample (keep away from light during this process). To distinguish the phagocytosis of Ox-LDL by Kupffer cells in the liver, we marked the localization of Kupffer cells using immunofluorescence (marked as F4/80). Since Ox-LDL have a red fluorescent probe Dil, we stained the nuclei of liver tissue with 4′,6-diamidino-2-phenylindole (DAPI) and observed it under a laser confocal microscope. The same parameters were used for each view.

### Detection of Ox-LDL and inflammatory factors in circulation

Two methods were used to take blood from the intraocular canthal vein of the mouse and the eyeball. To detect the content of Ox-LDL in the circulation, we collected blood from the intraocular canthal vein to obtain serum. After the mice were anesthetized with 3% chloral hydrate, the glass capillary was slowly rotated into the intraocular canthal vein and the blood was collected. To detect hs-CRP, TNF-α, MCP-1, and IL-6, we collected blood by taking the eyeballs directly with curved forceps. The blood sample obtained by the above method was allowed to stand at room temperature for three hours, centrifuged at 3500 r/min at room temperature for 15 min, and then the supernatant was transferred to EP tube and stored in a refrigerator at -80 °C. All these indicators in the circulation were detected by the corresponding ELISA kit.

### Western blot

To determine the effect of transduction in the liver, we detected the expression level of LOX-1 in the liver using Western blot. And we also examined the expression of Bax and Bcl-2 in the THP-1 to investigate the apoptosis of THP-1 after Ox-LDL stimulation. The specific steps were as follows. Total proteins were extracted from THP-1 or liver tissues using RIPA buffer, and the concentration of protein was quantified by a BCA protein assay kit according to the instructions. After the system was prepared and separated with 10% polyacrylamide SDS-PAGE gels, samples were then transferred to the polyvinylidene difluoride membrane. Subsequently, 5% skimmed milk powder were used to block the membrane at room temperature for 2 hours. After that, the membrane was incubated with specific antibody at 4 °C overnight and then were incubated with HRP-linked secondary antibody for 2 hours at room temperature. Finally, the bands were visualized with a chemiluminescence detection kit and quantified using Image Lab software system.

### Oil Red O staining

To investigate the progression of advanced atherosclerotic lesions, we determined the plaque of aorta and aortic root using Oil Red O staining. After mice were perfused from the heart with saline, we carefully separate the surrounding adipose tissue to obtain a complete aorta from the aortic arch to the bifurcation of the iliac artery. The aorta was stained with Oil Red O after fixation with 4% paraformaldehyde and digital images were taken with a digital camera. To further determined the plaque in aortic root, Oil Red O staining were also used on the sections of the aortic root (6 μm thick). Staining was assessed by optical microscopy and quantified (the results were expressed as the percentage of lesion area with lipid accumulation to the total lumen area) by Image-Pro Plus 6.0 software.

### HE and Masson

In order to determine the size of the necrotic core and the content of collagen fibers in the plaque, we performed hematoxylin-eosin (HE) staining and Masson staining on the sections of aortic root. The collected heart specimens were fixed in paraformaldehyde (4% in PBS), embedded in paraffin and then sectioned at 8 μm for HE and Masson staining. Images of the stained sections were visualized using a light microscope and quantified (the results were expressed as the percentage of cholesterol necrotic cores and the collagen fibers content to the plaque respectively) by Image-Pro Plus 6.0 software.

### Immunohistochemical and Immunofluorescence

The immunohistochemical method was employed to detect LOX-1 expression in the liver of the mice in two groups. And also, to determine whether the removal of circulating Ox-LDL is effective in stabilizing plaques, we used immunohistochemistry and immunofluorescence to detect the relevant components in the advanced plaque. We first detected the content of macrophages (marked as F4/80) in the advanced plaques by immunohistochemistry. As for immunofluorescence staining, we detected the localization of Kupffer cells (marked as F4/80) in the liver. And the expression of contractile vascular smooth muscle cells (marked as α-SMA) and synthetic vascular smooth muscle cells (marked as Vimentin) in the advanced plaques of the control and viral group were also determined using immunofluorescence staining respectively. The specific steps are as described in a previous study 17.

### Cell culture and intervention

*In vitro* culture of human mononuclear macrophage cell line THP-1, PMA induces the conversion to macrophages. Different concentrations of Ox-LDL (25, 50, and 100 mg/L) were provided to stimulate macrophages. CCK-8 kit was employed to detect cell proliferation. Hoechst33258 staining followed by fluorescence microscopy and light microscopy was utilized to observe foaming and apoptotic bodies of macrophages, and Western blot was deployed to detect the expression of apoptosis-related proteins Bax and Bcl-2.

### Statistical analysis

All data were collected from represent 3 to 5 independent replicates, and the results are expressed as mean ± standard deviation (SD). The data were statistically analyzed using Student's t-test or one-way ANOVA by SPSS 22.0 statistical software. P< 0.05 was considered statistically significant.

## Results

### LOX-1 was ectopically and functionally expressed in the liver as an Ox-LDL receptor

**Figure 1:**
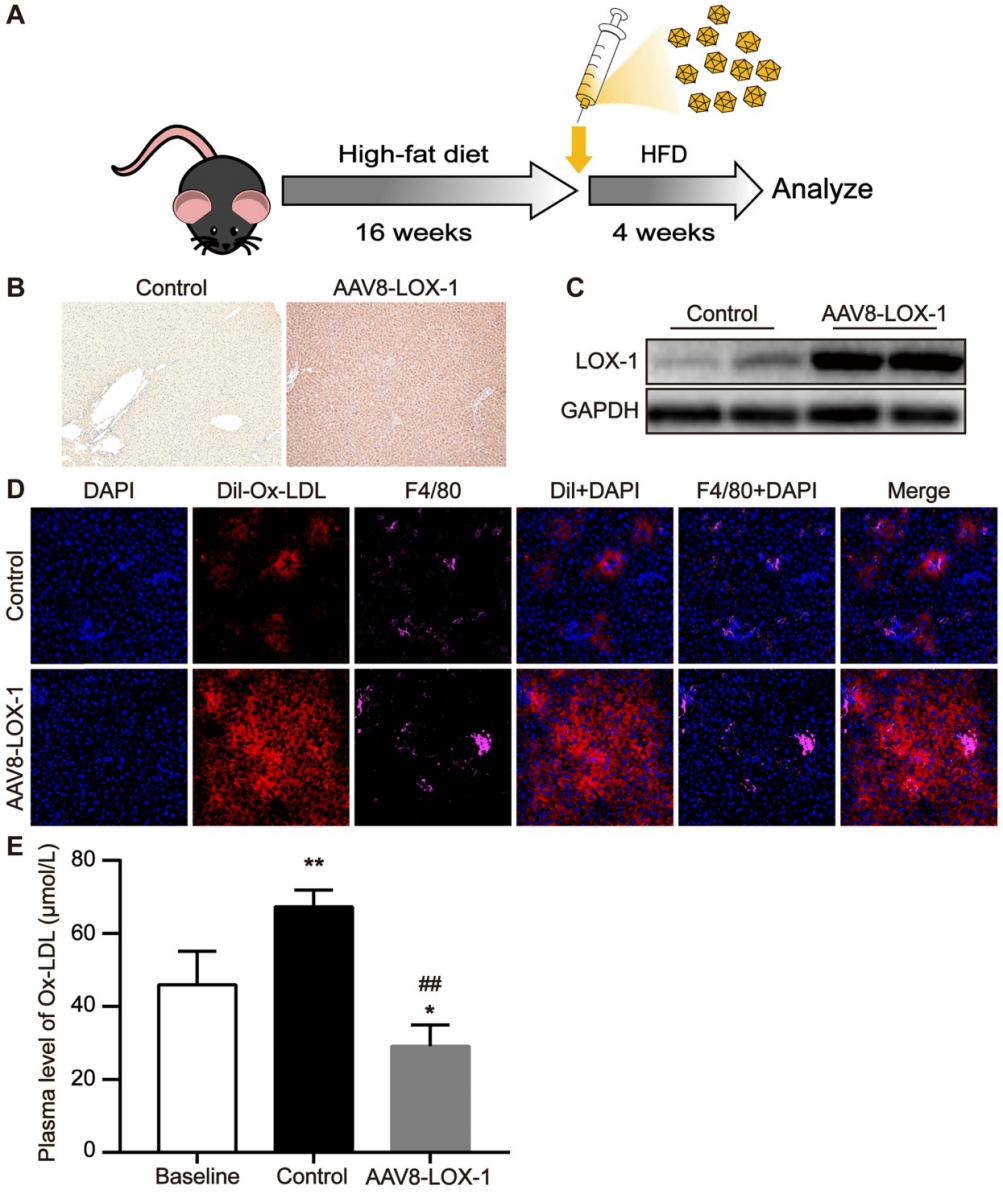
** LOX-1 was ectopically and functionally expressed in the liver as an Ox-LDL receptor. (A)** Schematic illustration of the experimental procedure. **(B)** Representative images of immunohistochemistry staining for the detection of LOX-1 in the liver (100x). Dark brown represents LOX-1 expression.** (C)** The protein expression of LOX-1 in liver of mice in study groups using Western blot analyses. **(D)** Representative images of fluorescence colocalization to determine the expression of F4/80 (represents Kupffer cells) and Dil-Ox-LDL in the liver after thirty minutes Dil-Ox-LDL injection (400x). Red fluorescence shows Dil-Ox-LDL, and purple fluorescence shows F4/80. **(E)** Plasma level of Ox-LDL was detected using ELISA. Values are expressed as mean ± SD (n = 10). *P<0.05 and **P<0.01 vs Baseline group, **^##^**P<0.01 vs. Control group.

First, AAV8 was injected into the virus group mice through the tail vein and then fed with high-fat diet. After feeding for four weeks, the mice were sacrificed and corresponding specimens were obtained (Figure 1A). Immunohistochemistry revealed that compared with control group, LOX-1 was extensively expressed in the liver of the mice in the virus group (Figure 1B), indicating that LOX-1 was ectopically expressed in the liver. Similarly, the results of western blot showed that the expression of LOX-1 in the liver of mice in the virus group gradually increased over time (Figure 1C), confirming that the liver of mice in the virus group successfully expressed LOX-1 after virus intervention. To test the function of LOX-1 in hepatocytes, we observed the phagocytosis of Dil-Ox-LDL in the liver frozen sections under laser scanning confocal microscope. Thirty minutes after Dil-Ox-LDL injection, we founded that scattered red-fluorescent appeared in the liver of control mice. Consistent with Van Berkel et al. reported 18, scattered red-fluorescent labels distributed around the Kupffer cells (marked as F4/80), which indicated that circulating Ox-LDL can be cleared by Kupffer cells in the liver under normal condition. Conversely, in the virus group mice, we found much more red-fluorescent showed in the hepatocytes and Kupffer cells. Thus, these results indicated that LOX-1 was ectopically and functionally expressed in the liver as an Ox-LDL receptor (Figure 1D). We also detected Ox-LDL content in the circulation. Consistent with the above results, the content of circulating Ox-LDL in the virus group was significantly reduced (Figure 1E). These data reflect that transfecting AAV8-LOX-1 not only enables the liver to successfully express LOX-1 but also enables LOX-1 to successfully play its role as an Ox-LDL receptor in hepatocytes.

### Continuous removal of circulating Ox-LDL inhibits rapid progression of advanced plaques

**Figure 2:**
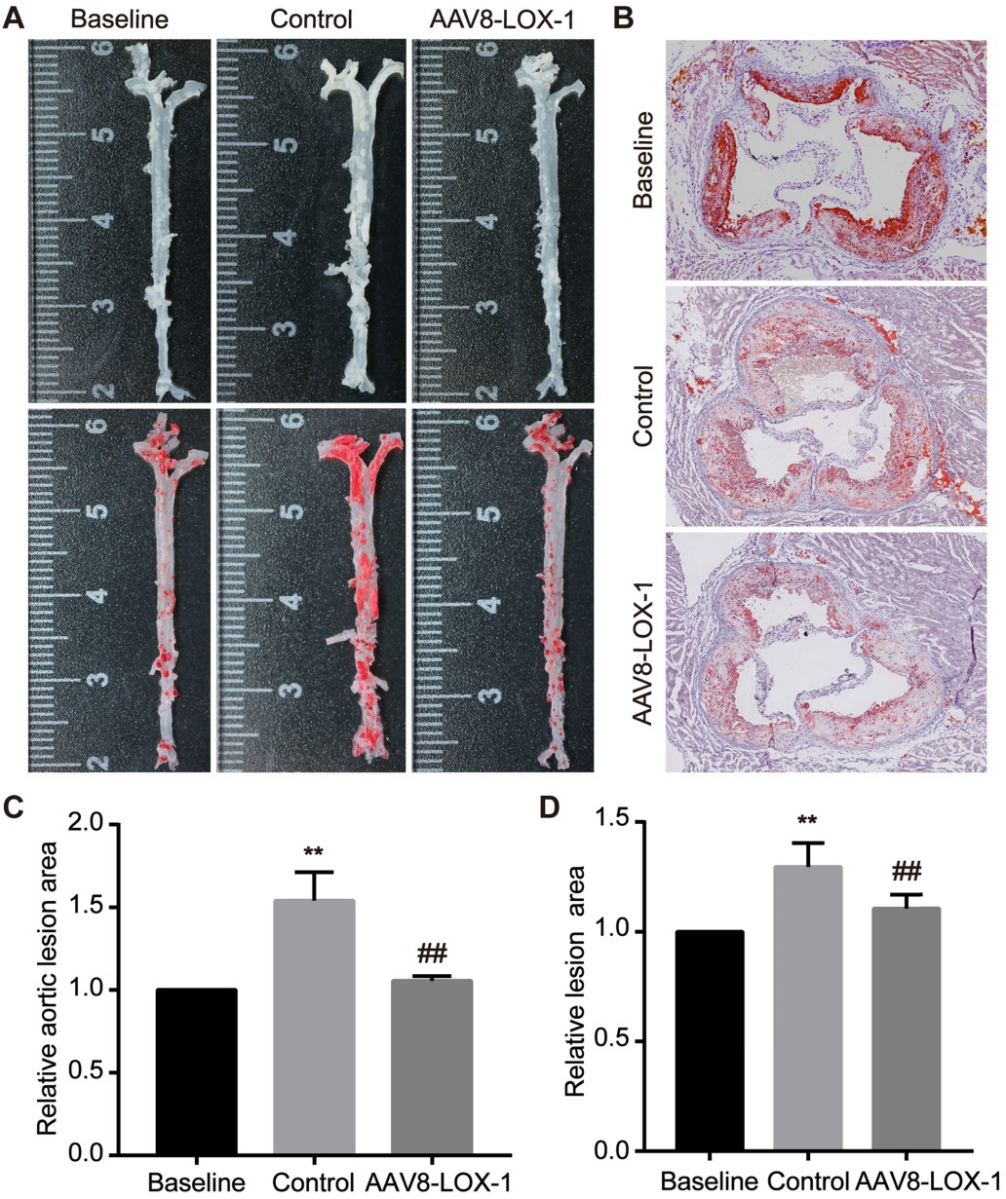
** Continuous clearance of circulating Ox-LDL inhibits the rapid progression of advanced atherosclerosis. (A)** Images of Oil Red O staining showing en face total aortas obtained from mice in study groups. **(B)** Photomicrographs of Oil Red O staining showing aortic root sections in study groups (100x). **(C)** The percent of the en face aortic arch area that was occupied by the positive area was analyzed. The baseline group is set as 1, and control and virus groups are compared with each other. **(D)** The ratio of the plaque area at the aortic root to the entire aortic root area in the indicated groups. The results are based on the baseline group 1 and the ratio of the remaining two groups. Values are expressed as mean ± SD (n = 10). **P<0.01 vs. Baseline group, **^##^**P<0.01 vs. Control group.

We performed corresponding tests to determine whether continuous elimination of circulating Ox-LDL through the liver in the late stage can inhibit advanced plaque progression. Oil Red O staining was applied to the aortas and aortic root sections of the mice to detect the size of atherosclerotic lesions. We found that compared with the baseline group, the area of ​​plaque in the aorta of the control group was 1.5 times that of the baseline group, while the virus group had no significant difference from the baseline group (set as 1) (Figure 2A and 2C). We also analyzed the results of aortic root and found that the area of lesions in the virus group was significantly relieved than that in the control group (Figure 2B and 2D). From these data, we can conclude that in the advanced stage of atherosclerosis, the disease progresses very rapidly, and a severe deterioration can occur in a short time. By continuous removal of circulating Ox-LDL, the rapid progress of advanced atherosclerosis can be obviously suppressed.

### Elimination of circulating Ox-LDL through liver can reduce necrotic cores and increase late plaque stability

**Figure 3:**
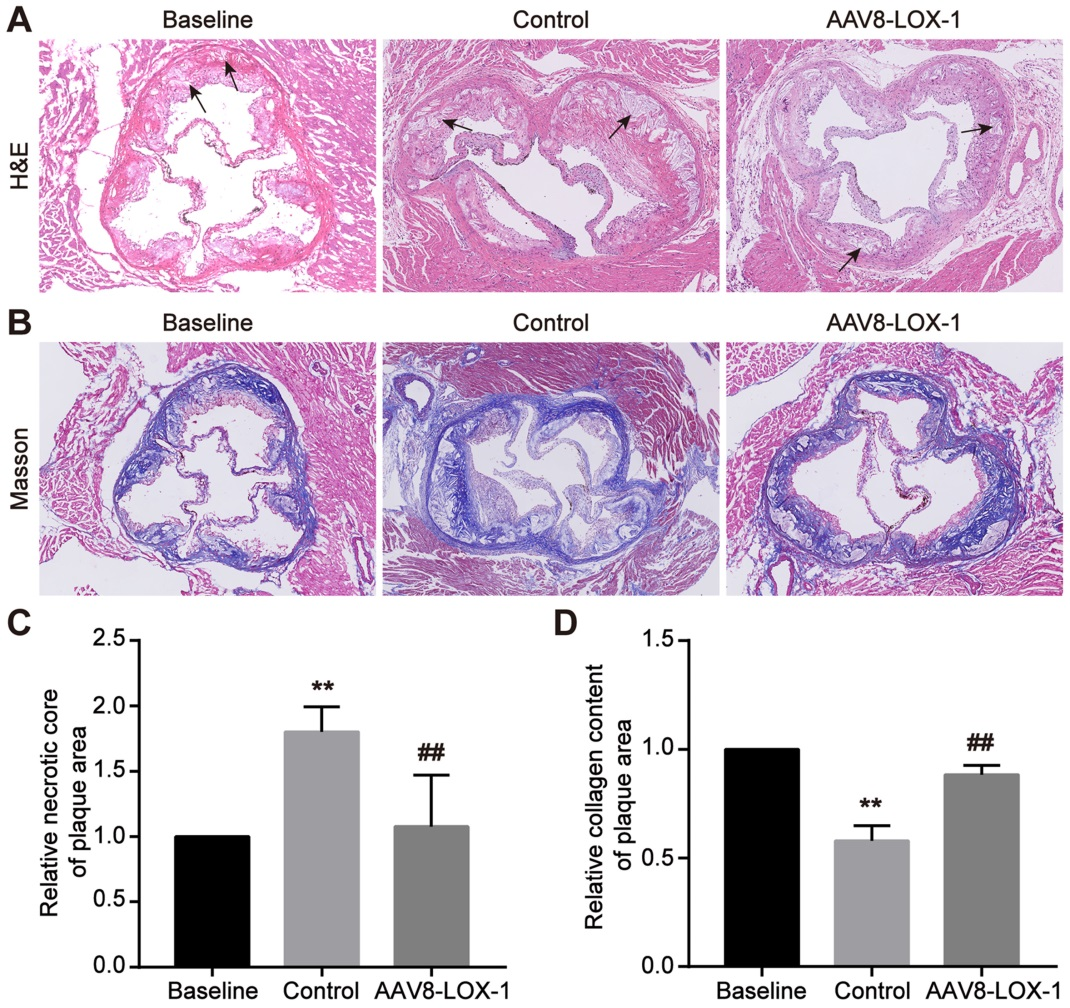
** Elimination of circulating Ox-LDL can reduce necrotic cores and increase late plaque stability. (A)** Representative images of HE staining for detecting the cholesterol necrotic cores in plaques in study groups (100x). The black arrow indicates the cholesterol crystal necrotic cores. **(B)** Representative images of Masson's trichrome staining to manifest the collagen content in plaques in study groups (100x).** (C)** The percent of the cholesterol necrotic cores area in plaque was analyzed. The baseline group is set as 1, and control and virus groups are compared with each other. **(D)** The statistical analysis of the collagen fibers content in the plaques of the three groups in Masson staining, with the baseline group as baseline 1, and the ratio of the remaining two groups to it. Values are expressed as mean ± SD (n = 10). **P<0.01 vs. Baseline group, **^##^**P<0.01 vs. Control group.

In the advanced stage of atherosclerosis, the vascular endothelial barrier was severely damaged. The completely destroyed endothelial barrier will not continue to provide protection, so advanced atherosclerosis progression is speedy. Through HE staining, we found that compared with the baseline group, the proportion of cholesterol necrotic cores in the plaque area of ​​the control group increased significantly, while the virus group was not much different from the baseline group (set as 1) (Figure 3A and 3C), which demonstrated that progression of advanced atherosclerotic lesions is very rapid, necrotic cores can expand rapidly in a short time, and continuous removal of circulating Ox-LDL can significantly reduce the proportion of necrotic cores. Simultaneously, Masson results indicated that the content of collagen fibers in plaques in the control group is significantly lower than that in the control group, and the fiber cap is extremely thin, while the collagen in the virus group is not much different from the baseline group (set as 1) (Figure 3B and 3D). It is inferred that continuous removal of circulating Ox-LDL in the advanced atherosclerosis can not only reduce the necrotic core but also maintain the collagen fiber content in the plaque and thus stabilize the advanced plaque.

### Elimination of Ox-LDL can reduce macrophages in advanced plaque and inhibit smooth muscle cell phenotype conversion

**Figure 4:**
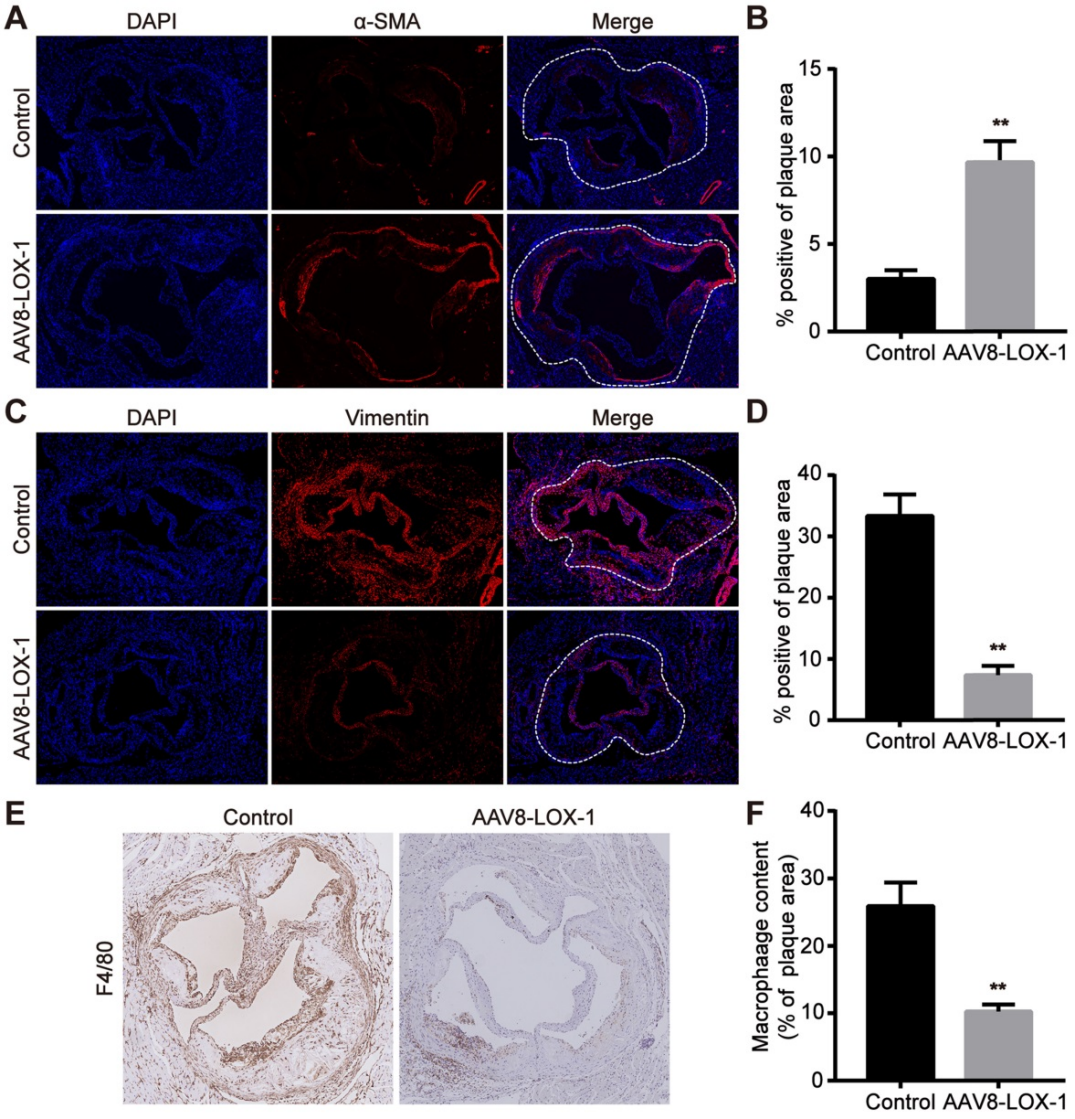
** Elimination of Ox-LDL can reduce macrophages in advanced plaques and inhibit smooth muscle cell phenotype conversion. (A-D)** Representative images of immnunofluorescence for the determination of α-SMA (A) and Vimentin (C) (100x). Red fluorescence shows α-SMA or Vimentin expression. Blue fluorescence shows nuclei. Quantitative analysis of the content of α-SMA (B) and Vimentin (D) in the plaque in virus and control groups. **(E)** Representative images of immunohistochemistry show the F4/80 content in plaques of virus and control groups (100x). **(F)** Quantitative analysis of F4/80 content in plaques of virus and intervention groups. Values are expressed as mean ± SD (n = 10). **P<0.01 vs. Control group.

As we all know, the foaming of macrophages and the conversion of smooth muscle cells are the most important factors leading to late plaque rupture. Hence, we tested the content of macrophages and smooth muscle cells in the plaques of control and virus groups. Previous studies have revealed that contractile smooth muscle cells marked by α-SMA beneficially affect the stability of plaques, while synthetic smooth muscle cells marked by Vimentin greatly promote the instability of plaques. The results of immunofluorescence showed that α-SMA of virus group was much higher than that of control group, and Vimentin was greatly reduced compared with the control group (Figure 4A-D) (Aortic root plaque has been marked with a dashed line). The immunohistochemistry results also showed that the content of macrophages (marked as F4/80) in plaques in the virus group was significantly reduced compared to the control group (Figure 4E,F). Based on these results, we reasonably speculate that the clearance of Ox-LDL not only reduced the content of macrophages in the late plaques but also inhibited the conversion of smooth muscle cells, which also helps stabilize advanced atherosclerotic plaque.

### Clearing Ox-LDL in the circulation can reduce MMP9 and Bax expression in the plaque

**Figure 5:**
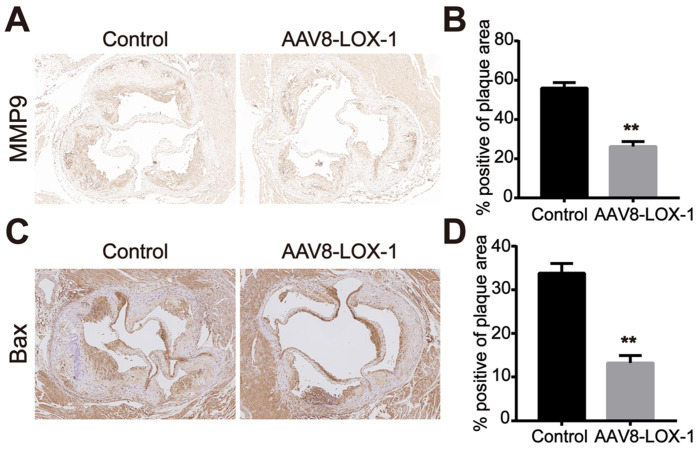
** Clearing Ox-LDL in the circulation can reduce MMP9 and Bax expression in the plaque. (A)** Representative images of immunohistochemistry for detecting MMP9 content in plaques of the two groups (100X).** (B)** The percentage of MMP9-positive area in plaques of the two groups in immunohistochemical staining was analyzed. **(C)** Representative images of immunohistochemistry for detecting the content of Bax in plaques of the two groups (100X). **(D)** The percentage of Bax-positive area in plaques of the two groups in immunohistochemical staining was analyzed. Values are expressed as mean ± SD (n = 10). **P<0.01 vs. Control group.

In the late stage of atherosclerosis, oxidized lipids rapidly deposit in blood vessels during circulation. Macrophages and smooth muscle cells phagocytose Ox-LDL through surface receptors. Its unsaturated phagocytic characteristics continue to induce the release of matrix metalloproteinases and foam necrosis. The results indicated that compared with the control group, the expression of matrix metalloproteinase MMP9 and apoptotic protein Bax in plaques of the intervention group was significantly reduced (Figure 5). Based on this, we speculate that the clearance of Ox-LDL can effectively reduce cell apoptosis and MMP9 secretion in advanced plaques.

### Clearing Ox-LDL in the circulation can alleviate the inflammatory stress in circulation

**Figure 6:**
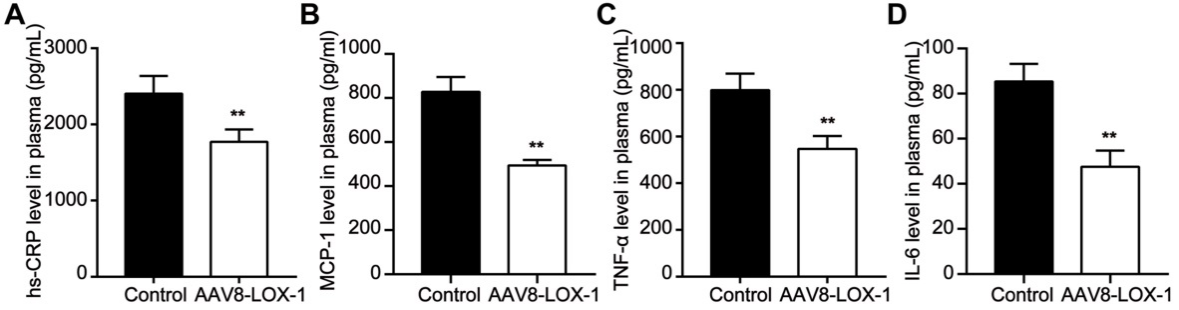
** Clearing Ox-LDL in circulation can alleviate inflammatory stress in circulation. (A-D)** Plasma level of hs-CRP **(A)**, MCP-1 **(B)**, TNF-α **(C)**, and IL-6 **(D)** were determined by using ELISA. Data are presented as mean ± SD (n=10). **P<0.01 vs. Control group.

Intravascular lipid deposition and induced inflammatory response chain run through the atherosclerotic lesions. Our study showed that compared with the control group, the content of hs-CRP, MCP-1, TNF-α, and IL-6 in the intervention group was significantly reduced (Figure 6). Accordingly, we speculate that clearing Ox-LDL in circulation can alleviate the inflammatory response.

### The cytotoxic effect of Ox-LDL in macrophages induces foaming and apoptosis of macrophages

**Figure 7:**
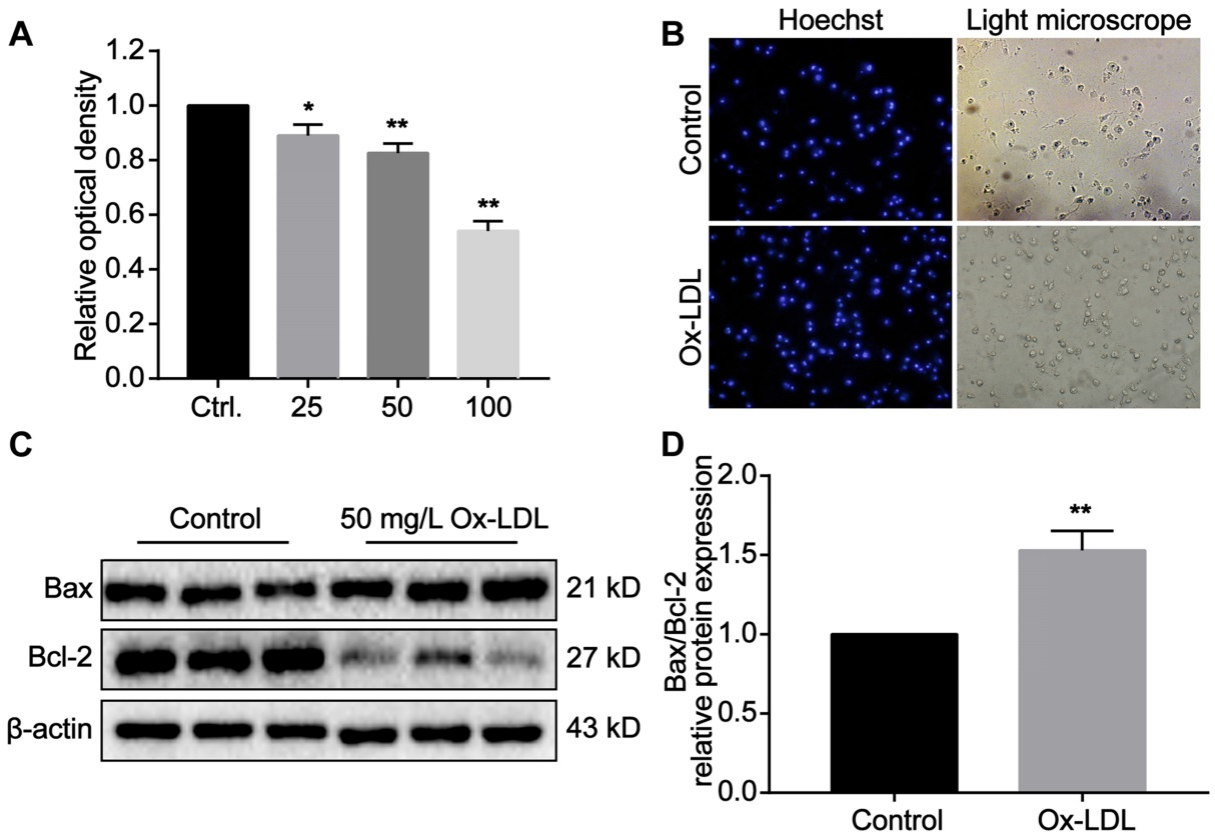
**The cytotoxic effect of Ox-LDL in macrophages induces foaming and apoptosis of macrophages. (A)** Ox-LDL affects the proliferation of macrophages in a concentration-dependent manner. The control group is set as 1. **(B)** Representative images of foaming changes after macrophages phagocytosis Ox-LDL (200X). **(C-D)** The protein expression of Bax and Bcl-2 in macrophage of study groups using Western blot analyses. The control group is set as 1. Data are presented as mean ± SD. *P<0.05 and **P<0.01 vs. Control group.

Macrophages phagocytose Ox-LDL through surface receptors. Due to its unsaturated phagocytic properties, Ox-LDL can induce foaming and apoptotic necrosis of macrophages. Our studies *in vitro* have revealed that the proliferation of macrophages is significantly inhibited as Ox-LDL concentration in the medium increases (Figure 7A). The light microscope indicated that the antennae of macrophages disappeared after phagocytosis of Ox-LDL. The cell outline was blurred, and the boundary between nucleus and cytoplasm was unclear (Figure 7B). Under stimulation of 50 mg/L Ox-LDL, Bax expression in macrophages increased, while Bcl-2 expression decreased. Compared with the control group, the ratio of the two is significantly different (Figure 7C,D). According to the above results, we reasonably speculate that reducing Ox-LDL can alleviate foaming and necrosis of macrophages, which consistent with our experiments *in vivo*.

**Figure 8:**
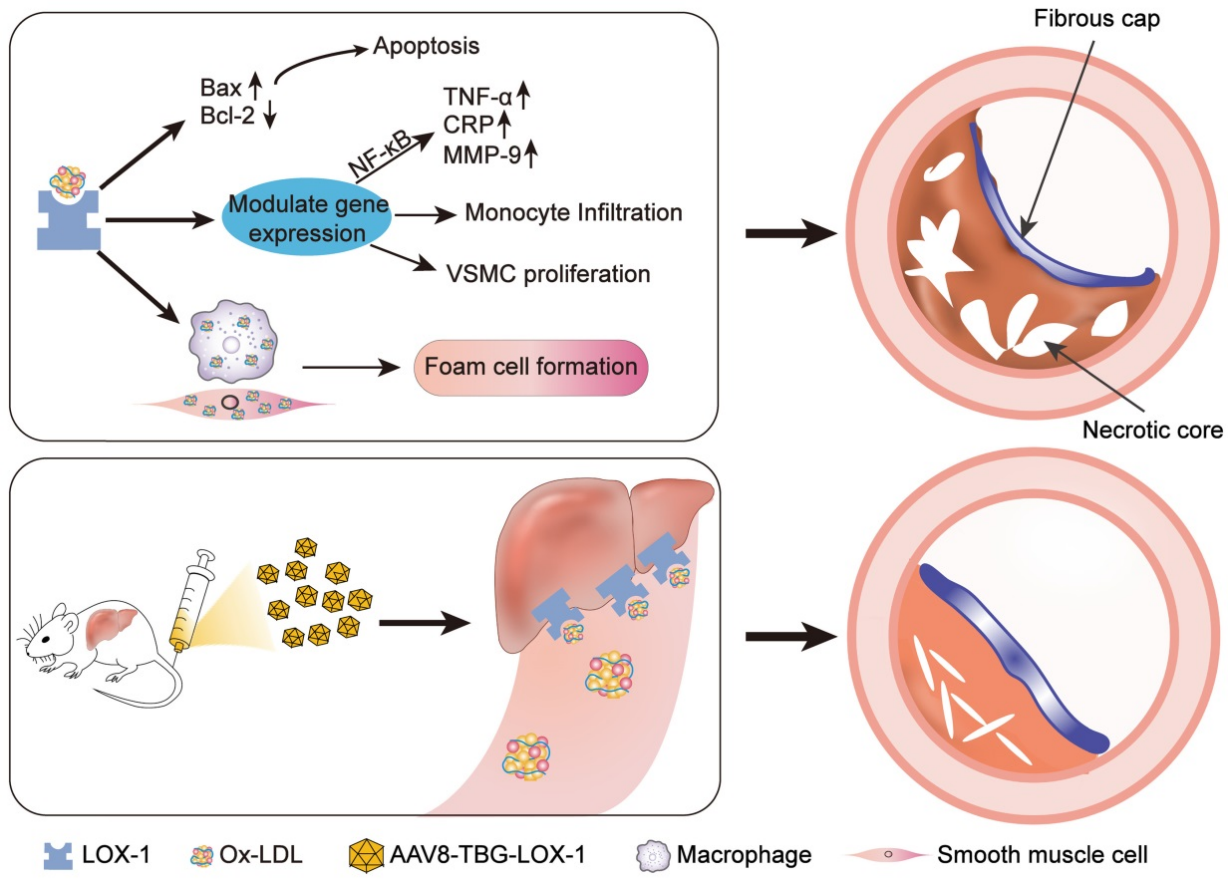
** Schematic illustration of the study.** Ox-LDL is phagocytosed by vascular wall endothelial cells, macrophages, and smooth muscle cells through LOX-1 receptors, causing a series of undesirable consequences: apoptosis, release of inflammatory factors, chemotactic migration of monocytes, smooth muscle cell migration, and type conversion. The above-mentioned adverse reactions can destruct vascular endothelial barrier, foaming and necrosis of macrophages and smooth muscle cells, degrade collagen fibers, and the continuous inflammatory response in the blood vessels ultimately leads to the formation of vulnerable plaques with huge necrotic core and low collagen fiber content. Here, gene editing technology was employed to allow hepatocytes to ectopically and functionally express LOX-1 as an Ox-LDL receptor, which dramatically reduced accumulation of Ox-LDL in circulation and inhibited the rapid progress of advanced atherosclerosis eventually.

In conclusion, we found that in the late stage, atherosclerotic lesions progress rapidly. The expansion of necrotic core and the thinning of fibrous cap will cause the plaque to become very fragile. However, removing circulating Ox-LDL through the liver can not only inhibit rapid progress of advanced atherosclerotic lesion but also stabilize advanced plaque and avoid acute cardiovascular events (Figure 8). On the basis of the current treatment with LDL reduction as the mainstream method, our study has laid a solid foundation for preliminary research on the effect of Ox-LDL clearing in the treatment of advanced atherosclerosis, and provides a new direction and concept for the clinical treatment of patients with advanced atherosclerosis. Therefore, we believe that our study has great potential research significance.

## Discussion

At present, acute cardiovascular events caused by atherosclerosis remain the leading cause of death in the world 19, although current lipid-lowering treatments have achieved remarkable results 20. However, according to research reports, applying lipid-lowering therapy cannot significantly reduce cardiovascular death probability in advanced atherosclerosis patients. Therefore, based on the current lipid-lowering treatment, exploring a new treatment direction will be able to better treat advanced atherosclerosis.

Many organs of the human body can absorb and degrade LDL, and the liver is the main organ. However, Ox-LDL formed after oxidative modification of LDL in plasma is mainly eliminated by the binding of macrophages in the monocyte-macrophage system and vascular endothelial cells through scavenger receptors on the surface 18. Different from hepatocyte cholesterol molecular metabolism receptors, macrophages and endothelial cell scavenger receptors did not down-regulate the receptor function after phagocytosis of Ox-LDL, causing excessive cholesterol deposition, starting the inflammatory response system, and causing apoptosis and pyrolysis 21. Under normal circumstances, the Ox-LDL produced in the body can be quickly cleared by Kupffer cells in the liver, but the continuous lipid metabolism disorder and inflammation in the process of atherosclerotic lesions cause the Ox-LDL in the circulation to continue to rise. It's difficult to eliminate all of it for the Kupffer cells in the liver, which eventually leads to the phagocytosis of vascular endothelial cells and triggers the destruction of endothelial barrier function. There have been more than 20 clinical studies on the correlation between Ox-LDL and atherosclerotic cardiovascular disease. After correction to exclude LDL cholesterol risk factors, After adjusting and excluding risk factors for LDL cholesterol, patients with elevated Ox-LDL have a 1.66-2.88-fold increase in the risk of atherosclerotic cardiovascular disease 22. Existing drugs are not enough to avoid the oxidation process of circulating and tissue lipids in lipid disorders, because elevated LDL is too easy to be oxidized and modified. Current lipid-lowering drugs have achieved sufficient LDL reduction effects, but how to remove Ox-LDL is still a difficult problem.

This research creatively explores new treatment directions beyond lipid-lowering and focuses on effective Ox-LDL removal. We propose to use liver's strong lipid metabolism capacity to continuously eliminate Ox-LDL of advanced atherosclerotic lesions. Our results suggests that clearing Ox-LDL not only inhibits the rapid advanced atherosclerosis progress but also reduces the necrotic core in plaque and stabilizes highly ruptured advanced plaque. Hence, we believe that eliminating Ox-LDL in the circulation is a critical treatment in addition to applying lipid-lowering drugs such as statins.

One of the main findings of this study is that continuous removal of circulating Ox-LDL in advanced atherosclerosis prevents rapid progression of plaque to a large extent. As we all know, once advanced plaque is established, the damaged endothelial barrier is unable to prevent the invasion of circulating LDL, Ox-LDL, and inflammatory cells. Rapid lipid deposition and inflammatory cells that continue to engulf lipids lead to a breakthrough in advanced atherosclerosis 23. We established advanced atherosclerotic plaques in male ApoE^-/-^ mice on a western high-fat diet for 20 weeks and found that the blood vessels that have lost the endothelial protection show rapid atherosclerosis progress 24. Simultaneously, the necrotic core increases, and collagen fiber content decreases. After Ox-LDL in circulation is continuously removed, this situation has been significantly curbed. Undoubtedly, we believe that our study provides an effective intervention direction for advanced plaque treatment and proves that Ox-LDL is harmful in advanced atherosclerosis.

Another major finding of this study is that continuous removal of circulating Ox-LDL in the late stage can improve the stability of late plaques. We all know that the most important features of fragile plaque are the huge necrotic core and extremely thin outer fibrous cap 25, 26. The combination of the two leads to plaque rupture, resulting in acute occlusive luminal thrombosis. Embolism caused sudden death from coronary heart disease 27-31. Our research shows that removing Ox-LDL from circulation can significantly reduce the necrotic core area in the plaque and increase the content of collagen fibers in the plaque. Therefore, we speculate that removing Ox-LDL can effectively inhibit the two major risks of plaque rupture. To further explore the changes in its composition, we learned that components of huge necrosis core are mainly macrophages and smooth muscle cells that undergo foaming and then apoptotic. We all know that during advanced atherosclerosis progression, macrophages play a very central role. Numerous macrophages not only self-foaming and apoptotic necrosis but also induce the apoptosis of smooth muscle cells in the plaque by secreting various cytokines to mediate the activation of multiple cell signals, such as Fas-L, NO, and TNF-α, leading to a rapid expansion of necrotic core in plaque. In addition, macrophages also decompose a large amount of extracellular matrix by secreting many matrix metalloproteinases, especially MMP-9, resulting in a sharp thinning of outer fibrous cap of plaque 32. Therefore, for multiple harmful effects of macrophages, we can say that fewer macrophages in plaque imply that the plaque is stable 33. Consistent with this, our study indicate that after clearing the circulating Ox-LDL in the late stage, macrophages in plaque have a very significant reduction compared with the control group, indicating that even in the late stage, the endothelial barrier is completely destroyed, removing Ox-LDL in circulation can significantly reduce the accumulation of macrophages in the circulation to the plaque, which greatly promotes the plaque stability. In addition, another important role that cannot be ignored in inducing advanced atherosclerotic plaque rupture is vascular smooth muscle cells. Switching of different phenotypes plays a crucial role in advanced atherosclerosis 34. Studies have shown that in the late stage of atherosclerosis, the vascular smooth muscle cells that migrate from media to intima change from a contractile type to a synthetic phenotype, not only secreting a large amount of MMPs and pro-inflammatory cytokines but also in the late plaque 50% of the foam cells are derived from this 11 35. In this conversion process, Ox-LDL is the culprit 36, 37. Consistent with this statement, our study revealed that after Ox-LDL was eliminated, synthetic VSMCs in ApoE^-/-^ mice plaques in the intervention group were significantly reduced, while smooth muscle cells with contractile phenotypes were significantly increased compared with those in the control group. These data indicated that clearance of Ox-LDL can inhibit VSMC phenotype conversion and contribute to maintenance of contractile phenotype, stabilize late plaques to a large extent, protect the maintenance of the outer fibrous cap of the plaque and avoid the rapid thinning of the fiber cap due to secretion of numerous destruction molecules by smooth muscle cells of the synthetic phenotype, which plays a beneficial role in stabilizing the late plaque. In addition, our research showed that the expression of apoptotic protein Bax and matrix metalloproteinase MMP9 in the plaque of the intervention group was significantly reduced, confirming our hypothesis that Ox-LDL elimination in the circulation reduced apoptosis and MMP secretion of macrophages and smooth muscle cells in the plaque.

Another major hypothesis for advanced atherosclerosis is that excessive inflammatory stress in the late stage of atherosclerosis is one of the leading cause of plaque rupture 38, 39. Interestingly, we found that after clearing Ox-LDL, mice in the intervention group had significantly lower circulating levels of MCP-1, IL-6, TNF-α, and hs-CRP compared with the control group, indicating that the elimination of Ox-LDL can also inhibit the inflammatory stress in mice, which is undoubtedly beneficial for stability of plaques.

All in all, our research shows that the plaque progresses abnormally rapid in the late stage of atherosclerosis. Using the current mature gene editing technology, the liver cells can express the Ox-LDL specific receptor LOX-1 by transfecting the liver with AAV8 containing a specific promoter. Then the liver can successfully bind and continue to clear the circulating Ox-LDL, which effectively inhibit the rapid progress of advanced atherosclerotic plaque, reduce the necrotic core, and enhance the stability of the plaque. We believe that this study provides a novel and feasible treatment method for the treatment of advanced atherosclerosis in addition to lowering lipids.

## Abbreviations

Ox-LDL: oxidized low density lipoprotein
AAV8: adeno-associated virus type 8
ApoE^-/-^: apolipoprotein E knockout
LOX-1: lectin-like oxidized low density lipoprotein receptor 1
LDL: low-density lipoprotein
HE: hematoxylin-eosin
Bax: Bcl2-associated X
Bcl-2: B-cell lymphoma-2
hs-CRP: hypersensitivity C-reactive protein
TNF-α: tumor necrosis factor-α
MCP-1: monocyte chemotactic protein-1
IL-6: interleukin-6
DAPI: 4′,6-diamidino-2-phenylindole
BCA: bicinchoninic acid
α-SMA: α-smooth muscle actin
MMP9: matrix metalloproteinase 9
VSMC: vascular smooth muscle cell
